# Selection of Reference Genes for qPCR- and ddPCR-Based Analyses of Gene Expression in Senescing Barley Leaves

**DOI:** 10.1371/journal.pone.0118226

**Published:** 2015-02-27

**Authors:** Agnieszka Zmienko, Anna Samelak-Czajka, Michal Goralski, Ewa Sobieszczuk-Nowicka, Piotr Kozlowski, Marek Figlerowicz

**Affiliations:** 1 Molecular and Systems Biology Department, Institute of Bioorganic Chemistry, Polish Academy of Sciences, Poznan, Poland; 2 Institute of Computing Science, Faculty of Computing, Poznan University of Technology, Poznan, Poland; 3 Department of Plant Physiology, Faculty of Biology, Adam Mickiewicz University, Poznan, Poland; University of Western Sydney, AUSTRALIA

## Abstract

Leaf senescence is a tightly regulated developmental or stress-induced process. It is accompanied by dramatic changes in cell metabolism and structure, eventually leading to the disintegration of chloroplasts, the breakdown of leaf proteins, internucleosomal fragmentation of nuclear DNA and ultimately cell death. In light of the global and intense reorganization of the senescing leaf transcriptome, measuring time-course gene expression patterns in this model is challenging due to the evident problems associated with selecting stable reference genes. We have used oligonucleotide microarray data to identify 181 genes with stable expression in the course of dark-induced senescence of barley leaf. From those genes, we selected 5 candidates and confirmed their invariant expression by both reverse transcription quantitative PCR and droplet digital PCR (ddPCR). We used the selected reference genes to normalize the level of the expression of the following senescence-responsive genes in ddPCR assays: *SAG12, ICL, AGXT, CS* and *RbcS*. We were thereby able to achieve a substantial reduction in the data variability. Although the use of reference genes is not considered mandatory in ddPCR assays, our results show that it is advisable in special cases, specifically those that involve the following conditions: i) a low number of repeats, ii) the detection of low-fold changes in gene expression or iii) series data comparisons (such as time-course experiments) in which large sample variation greatly affects the overall gene expression profile and biological interpretation of the data.

## Introduction

The determination of gene expression helps to dissect a gene’s functions, and real-time quantitative PCR (qPCR) is a sensitive and accurate method of measuring the levels of gene expression in small- to medium-scale studies. The accuracy of the qPCR measurement, however, relies on proper experiment design, especially on the selection of well-validated reference genes, the expression of which is generally insensitive to the conditions tested [1]. Any variation in the transcript level measured for reference genes would therefore reflect technical bias that was introduced during the experiment setup, and this information should be used for data normalization. A substantial number of qPCR analyses described in the literature are based on 1 reference gene only. However, it has been known for some time that whereas some genes are known to be stably expressed in many conditions, they still can be differentially regulated by specific developmental or environmental stimuli in particular tissues or organisms [2–5]. As a consequence, the authors of the MIQE guidelines (MIQE—Minimum Information for Publication of Quantitative Real-Time PCR Experiments) have recently proposed that the selection and testing of candidate genes should be an integral part of any new qPCR experiment setup [1]. Additionally, averaging data for 3–4 reference genes is highly recommended.

The recently developed droplet digital PCR (ddPCR) technology offers several advantages that conventional qPCR lacks [6]. In this method, the sample is diluted and divided into multiple aliquots (∼20,000 droplets in the case of the BioRad QX200 system) and subjected to endpoint PCR. The quantification of DNA/RNA molecules relies on the ability of ddPCR system to determine the number of target molecules by Poisson statistical analysis of “positive” (containing amplified target) and “negative” (no amplified target detected) droplets [7], [8]. Currently, the main postulated usage of ddPCR is detecting copy number variations and rare mutations in genomic DNA sequences. However, it is also becoming an increasingly useful tool in gene expression analysis [9–13]. The advantages of ddPCR over qPCR are as follows: lower sensitivity to factors that partially inhibit target gene amplification; robustness to variations in PCR efficiency; absolute quantification of the target without requiring a standard curve; and a linearity of the process, which allows the detection of small fold changes in gene expression. It has been shown recently that the correlation between qPCR and ddPCR results is generally good, although the latter technique shows higher precision and reproducibility [13–15]. Because of the ability of ddPCR to absolutely quantify the number of molecules present within a sample, the use of a reference gene is not obligatory in ddPCR. It should be stressed, however, that the outcome of the ddPCR assay still relies on precise quantification of the input samples and can be affected by all of the technical issues associated with the reverse transcription step [16]. Thus, the application of the reference genes with stable expression may be beneficial for ddPCR data normalization, especially when a low number of replicates are assayed or the sample quality is highly variable.

Our research interest focuses on the process of leaf senescence, which is a tightly regulated developmental or stress-induced process. It occurs in conjunction with dramatic changes in cell metabolism and structure and eventually leads to chloroplast disintegration, leaf protein breakdown, internucleosomal fragmentation of nuclear DNA and ultimately cell death [17–19]. Because of the global and intensive changes that occur in the transcriptome of the senescing leaf and the resultant difficulties in selecting stable reference genes, measuring a time-course gene expression pattern in this system is challenging. Recent studies have used qPCR-based analyses of gene expression in senescing leaves, usually utilizing 1 or 2 reference genes: actin-1 or α-tubulin for dark-induced senescence in rice [20]; actin-2, UBQ10 or UBQ5 + S16 for dark-induced senescence in Arabidopsis [21–23]; EIF4A1 for natural senescence in Arabidopsis [24]; actin and GAPDH for natural senescence in clover [25] and 18S rRNA and Splicing factor 2 (as a control) in barley [26]. These reference genes are considered to be “universal”, but only in 2 of the above studies, the choice of genes was supported by initial analysis of gene expression stability under the conditions tested [23], [26]. Recently, several candidate reference genes were evaluated in a sunflower leaf senescence model with the use of geNorm, BestKeeper and LMModel tools [27]. Again, however, the selection process was restricted to a set of “typical” candidates. As a consequence, qPCR studies of plant leaf senescence still rely on a narrow list of arbitrarily selected reference genes.

Microarray databases are good resources of gene expression data for the initial selection of candidate reference genes. The literature provides several examples of successful utilization of microarray data for such a purpose [28–31]. Additionally, there is a freely available RefGenes tool that analyzes user-selected microarray data from the Genevestigator database and reports a fixed number of 20 candidate genes with the most stable expression levels [32]. However, the Genevestigator database does not cover all conditions of interest; therefore, the search for reference genes often relies on suboptimal datasets, and the resulting candidates may fail further validation. Having faced problems using both the literature and RefGenes for the selection of good reference genes to analyze barley leaf senescence, we decided to perform microarray-based gene screening to discover genes with expression levels that are least affected by the senescence process. We created a list of 181 candidates that can serve as a useful resource for the selection of suitable reference genes. We validated the expression stability of 5 genes by qPCR and ddPCR methods. Finally, we applied reference gene-based normalization to quantify transcripts of 5 senescence marker genes using ddPCR. Our results prove that the reference genes approach is beneficial for reducing data variation in ddPCR assays.

## Materials and Methods

### Plant material and senescence induction experiments

Barley (*Hordeum vulgare* L. ‘Nagrad’) seedlings were grown for 7 days in soil under controlled conditions (day/night 16/8 h, 23°C, light intensity 150 μmol m-2 s-1, 60% humidity). The material for the day 0 sample was then collected, and the senescence process was induced by placing the seedlings in the dark. Leaves were collected at day 3, day 5, day 7, day 10 and day 12, and the samples were named accordingly. Samples from 3 biological replicates (independent cultivations) were obtained, and each sample was a pool of ∼15 plants.

### RNA extraction and cDNA synthesis

Total RNA was extracted from frozen barley leaves with spin-columns (RNeasy Plant Mini Kit, QIAGEN) and DNase-digested with TURBO DNA-free kit (Ambion) according to the manufacturers’ standard protocols. RNA quality was determined using Nanodrop 2000 and 2100 Bioanalyzer (Agilent). All of the samples used for the study were pure (A^260^/A^280^ ≥ 1.9; A^260^/A^230^ ≥ 2) and showed no visible signs of degradation. 1 μg RNA was used for reverse transcription in 20-μl reactions using SuperScript III reverse transcriptase (Invitrogen) and random pentadecamers. The reactions were carried on for 1 h at 50°C and stopped by incubation for 5 min at 85°C.

### Barley microarray hybridization and analysis

Labeled cRNA samples were prepared from 200 ng RNA each, using Quick Amp Labeling Kit (Agilent) and hybridized to Barley Gene Expression Microarrays, 4x44K (Agilent) according to a common reference design. Cy5-labeled samples of interest (Day 0, Day 3, Day 7 and Day 10, biological replicates a-c) were each hybridized against a Cy3-labeled common reference (RNA pool of all samples) on a total of 12 microarrays. All of the hybridization, washing and drying steps were performed in A4x44k Quad Chambers in an HS 4800 Pro (Tecan) automatic hybridization station according to the manufacturer’s guidelines regarding Agilent microarrays treatment. A Gene Expression Hybridization Kit (Agilent) and Gene Expression Wash Buffer Kit solutions (Agilent) were used for the hybridization and washing steps, respectively. The intensity data were collected with 4200AL GenePix scanner and GenePix Pro 6.1 software. Each microarray was scanned at low and high saturation levels, and spot intensities were merged after within-array normalization step. The microarray data were analyzed using a R/Bioconductor limma package [33]. Bayesian linear modeling, implemented in limma, was used for the evaluation of differential gene expression during senescence in comparison with Day 0. Statistically significant results were selected at F-p values < 0.0005, after applying Benjamini and Hochberg's method to control the false discovery rate. The data were deposited in Gene Expression Omnibus repository and are accessible through GEO Series accession number GSE62539 (http://www.ncbi.nlm.nih.gov/geo/query/acc.cgi?acc=GSE62539) [34].

### Natural senescence microarray data sets

Processed gene expression data for the experiment analyzing the natural senescence of barley flag leaves were taken from Christiansen and Gregersen [26]. Normalized expression data from an Arabidopsis leaves natural senescence experiment described in [35] were downloaded from the GEO repository, accession GSE22982. Bayesian linear modeling [33] was applied to evaluate differential gene expression between consecutive time-points (disregarding potential diurnal changes in gene expression; see description of the dataset in the original paper for details).

### Barley probe annotation

Barley Gene Expression Microarray probe sequences were used for *megablast* search against the GenBank *nr* nucleotide database, limiting the search to *Hordeum vulgare* sequences, e value < 0.001 and plus/plus orientation of the query-subject match. The probes with no matches were then used as queries against the *est* database, with the same limits. To annotate barley microarray results, the best hits from both searches were used in a *blastx* search against Arabidopsis protein model sequences (TAIR10), with an e value < 0.001 threshold.

### qPCR assays

Six-point standard curves for each gene were prepared from a serial dilution of pooled cDNA, as described in [36]. Gene expression was assayed in Rotor-Gene Q (Qiagen), 72-well rotor, using FastStart SYBR Green Master kit (Roche), 3 μl 35×diluted cDNA samples and the recommended thermal profile (40 cycles). This was followed by a melt curve analysis and gel electrophoresis of the qPCR products. For each target gene, all of the cDNA samples, standards and no template controls (reactions without cDNA) were assayed in triplicate in a single run. The standard curve calculation and data analysis was performed with Rotor-Gene Q software (Qiagen). The following primers were designed with Primer-BLAST: for Ref A (125 bp amplicon), forward: 5’-CCTTTGCTCAGGGCTTGCCAGA-3’ and reverse: 5’- AACGCGGATCCTATGATGGCAC-3’; for Ref B (193 bp amplicon), forward: 5’- AGAGCGGTTTGTGTTCTCCTTGG-3’ and reverse: 5’- GGGGGTGGGCAACAACGCTT-3’; for Ref C (110 bp amplicon), forward: 5’- ATACCCTGCCTTCACTGCGTGT-3’ and reverse: 5’- TGAGACCAGGCTTCCTTGGGATT-3’; for Ref D (156 bp amplicon), forward: 5’- TGTCTGCGGGTAGAAGAAGTGGA-3’ and reverse: 5’- GCTGAGTTACTCCCCTCACTCACA-3’; and for Ref E (108 bp amplicon), forward: 5’- CCACAAGTAGGCACTTCAGGTAG-3’ and reverse: 5’- CCTGTGAGATGTGGAATCCGCTGC-3’.

### ddPCR

The ddPCR assays were performed according to [37]. Briefly, each of the 20-μl reactions contained 1× EvaGreen ddPCR Supermix (Bio-Rad, Hercules, CA, USA), 200 nM gene-specific primers and 2 μl of the cDNA sample (∼100 ng). The following primers for SAG 12 were designed with Primer-BLAST: forward: 5’- CCCATCTGCAACACCAAGC-3’ and reverse: 5’- AATGCCAGAGAAAGCACCTAT-3’ (111 bp amplicon). Primers targeting ICL, AGXT, CS and RbcS were designed based on [26]. All of the other primers were the same as for the qPCRs. Each reaction was mixed with 70 μl of Droplet Generation Oil (Bio-Rad), partitioned into 14,000–17,000 droplets in QX200 Droplet Generator (Bio-Rad), transferred to 96-well plates (Eppendorf) and sealed. The PCRs were performed in a C1000 Touch Thermal Cycler (Bio-Rad) with the following cycling conditions: 1× (95°C for 5 min), 40× (95°C for 30 s, 57°C for 30 s, 72°C for 45 s), 1× (4°C for 5 min, 90°C for 5 min) with 2°C /s ramp rate. Immediately following end-point amplification, the fluorescence intensity of individual droplets was measured with the QX200 Droplet Reader (Bio-Rad). The data analysis was performed with QuantaSoft droplet reader software (Bio-Rad). Positive and negative droplet populations were detected automatically or (in case of 2 genes) manually on two-dimensional graphs. The target mRNA concentrations were calculated using the Poisson statistics [15] and background-corrected based on the no template control data. The absolute transcript levels were initially computed in [copies/μl PCR] and in [copies/ng cDNA]. In the latter case, the correction for the input cDNA amount was applied to each sample. The concentrations of cDNA (measured with Nanodrop 2000) were comparable between the samples used for ddPCR, which indicated similar efficiency of the reverse transcription step. Also, the [copies/μl PCR] and [copies/ng cDNA] measures were highly correlated (the linear correlation coefficient R^2^ was 0.973–0.985 for reference genes and 0.984–0.999 for the senescence marker genes). Throughout the article we present [copies/μl PCR] values.

## Results and Discussion

### Microarray-based experiments reveal multiple genes with stable expression in the course of dark-induced leaf senescence in barley

We have studied the process of senescence of barley leaves using a well-established experimental model [38–41]. In this model, the senescence process is induced by continued incubation of the seedlings in darkness. All of the data presented in this work were obtained from material collected in three independent, time-separated leaf senescing experiments (biological replicates). In each replicate, plant samples for the expression analysis were collected before senescence induction (Day 0) and after 3, 5, 7, 10 and 12 days of incubation in the dark. At the last stage (Day 12), the leaves turned yellow, and expansive changes were visible in the cell ultrastructure (S1 Fig.).

We investigated senescence-associated changes at selected time-points (Day 3, 7 and 10 *versus* Day 0) using Agilent 4x44k barley oligonucleotide microarrays. Of 43,603 unique oligonucleotide probes present on the microarray, 2,096 exhibited differential gene expression (at least two-fold change at any time point, with moderated F-statistic-p value<0.0005). Of these, 746 of them also revealed at least a two-fold change between the early and late senescence stage (Day 3 versus Day 10). (The detailed characterization of the changes in barley transcriptome during dark-induced leaf senescence will be described in a separate paper). To identify the genes with stable expression throughout the whole senescence period, we adopted the analysis scheme proposed by Czechowski et al. [28]. We searched for genes that showed a less than 20% expression change at all of the analyzed time-points and with mean expression levels (reflected by the normalized signal intensity A_mean_) within the range of typical expression of differentially expressed genes (the potential genes of interest). Of 2,681 genes meeting these criteria, 181 displayed very low signal intensity variation across individual hybridizations (CV_A_ < 0.01). This set (S1 Table) serves as a starting list of promising candidate reference genes that can be further subjected to individual selection. For the purpose of functional annotation, we provide the GenBank ID for each target sequence as well as a description of the best Arabidopsis protein match, when available.

We next selected 5 candidates from the list for verification, focusing on genes represented by abundant sequence data in the NCBI/Unigene database. The genes were also weakly responsive to various treatments, which was verified with the use of the following online tools: Genevestigator/Perturbation (54 gene-chip experiments) and PlexDB /GeneOscilloScope (108 gene-chip experiments). For the purpose of this work, we named the genes Ref A—Ref E (Table 1). Homology-based annotations indicate that Ref A (GenBank ID:AK356185) codes for a cytosolic enzyme pyruvate kinase, which may be involved in glycolysis; Ref B (GenBankID: AK252899) codes for nuclear NADH dehydrogenase [ubiquinone] 1 alpha subcomplex assembly factor 3-like; Ref C (GenBankID: AK373172) codes for a chloroplastic thioredoxin of M-type; Ref D (GenBankID: AK356259) codes for RING zinc finger domain superfamily protein; and Ref E (GenBank ID: GH223017) codes for a nuclear protein of unknown function. The genes are categorized in distinct Gene Ontology terms, and their products are likely present in three different cellular compartments: cytosol, mitochondrion or plastid. We have not found any evidence of their functional relation to one another. We may therefore assume that the independent confirmation of expression stability of each gene during senescence will prove the correctness of our reference gene selection process.

**Table 1:** Candidate reference genes with stable expression during leaf senescence in barley.

**Gene**	**Sequence data**		**Probe ID**		**Best Arabidopsis match**	
	**Nucleotide**	**Unigene (No. of sequences)**	**Agilent 4x44k microarray**	**Affymetrix 22k gene chip**	**TAIR locus**	**Annotation**
Ref A	AK356185	Hv.2627 (185)	A_13_P223939	Contig2551_s_at	AT3G52990	Pyruvate kinase family protein, cytosolic
Ref B	AK252899	Hv.20290 (13)	A_13_P073561	Contig14457_s_at	AT3G60150	NADH dehydrogenase [ubiquinone] 1 alpha subcomplex assembly factor 3, mitochondrial
Ref C	AK373172	Hv.9031 (15)	A_13_P149150	Contig9163_at	AT3G15360	Thioredoxin M-type, chloroplastic
Ref D	AK356259	Hv.10475 (27)	A_13_P220544	Contig8615_at	AT3G15070	RING/U-box superfamily protein, cytosolic
Ref E	GH223017	Hv.23706 (12)	A_13_P264922	Contig8843_at	AT4G13530	Unknown protein, nuclear

We note here that 18S rRNA and Splicing factor 2 genes previously used as normalization control and internal control, respectively, in barley flag leaf senescence experiment by Christiansen and Gregersen [26] are not represented by any probe in the sense orientation on Agilent 4x44k barley microarray. For this reason they were not considered as potential reference genes in the current study. It should be mentioned, however, that the Splicing factor 2 gene expression was found to be weakly affected by various treatments, similarly to our selected candidates (S2 Fig.). 18S rRNA gene showed much higher expression variability.

### qPCR assays confirm expression stability of the candidate reference genes during leaf senescence

**Fig 1:**
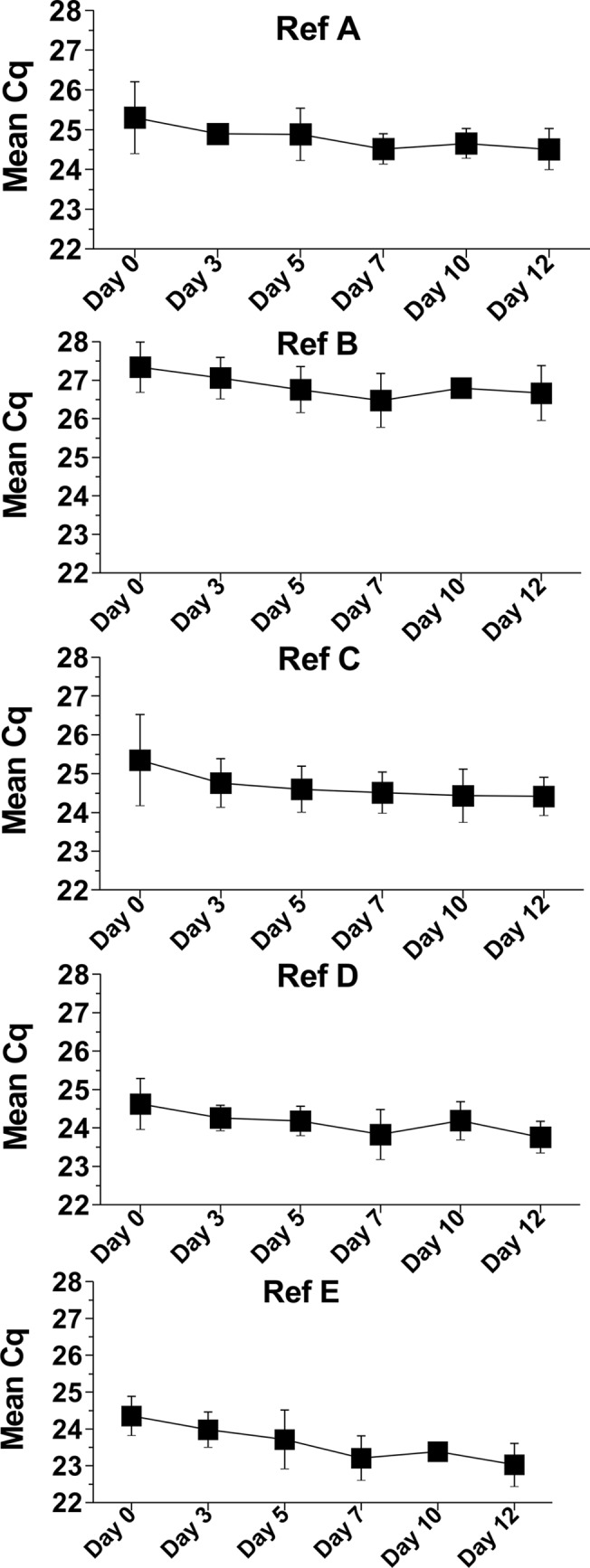
The expression of Ref A—Ref E genes during leaf senescence, presented as Cq values in qPCR assay. Each Cq is the mean from three biological replicates, and each replicate Cq value is averaged from three technical replicates. The whiskers present the distribution of the Cq values between the biological replicates. The scales are identical on all plots.

To confirm the stability of the expression of selected reference genes, we designed primers for each mRNA in such a way that they preferentially matched the region covered by Agilent microarray probes. Gene expression during leaf senescence was profiled using qPCR. The assay included 6 experimental time-points (Days 0 to 12, see above) and 3 biological replicates, a-c (18 samples total); each sample was run in triplicate. We observed very low variability of qPCR measurements for all of the tested genes (Fig. 1). The Cq value measurements (corresponding to the level of target mRNA in the samples) differed by a max. of 0.3–1.8 between the biological replicates for one time-point (see whiskers on the plots on Fig. 1), and the mean Cq values differed by a max. of 2.2–2.4 across time points. We also calculated the relative stability of gene expression using a dedicated geNorm software [42], using the Cq values of individual samples as input. This software ranks genes according to the stability of their expression and reports the M value, which should be less than 1.5 for suitable reference genes, according to the authors’ guidelines. The M values for Ref A—Ref E genes were, respectively, 0.022, 0.021, 0.025, 0.019 and 0.026, proving the stability of their expression in the tested conditions (Table 2). The geNorm software also calculates pairwise variation (V) between subsequent normalization factors for increasing numbers of reference genes in order to find the optimal gene number (i.e., the number for which the addition of another reference gene does not significantly reduce the assay variability further (V<0.15 is a recommended threshold)). According to this analysis, any two of our reference genes are sufficient to provide a reliable normalization factor (in each case, V <0.006).

**Table 2:** Expression stability of candidate reference genes calculated with geNorm.

Pairwise M values					
	Ref A	Ref B	Ref C	Ref D	Ref E
Ref A		0.020	0.020	0.018	0.028
Ref B	0.020		0.028	**0.014**	0.023
Ref C	0.020	0.028		0.023	0.030
Ref D	0.018	**0.014**	0.023		0.022
Ref E	0.028	0.023	0.030	0.022	
**Average gene M values**					
	0.022	0.021	0.025	0.019	0.026
**Rank**					
	II	**I**	III	**I**	IV

Genes with M value ≤ 1.5 are considered highly stable across analyzed samples [42]

The geNorm analysis proved that all of the candidate genes selected from the microarray data were suitable for qPCR data normalization in the barley leaves senescence experiment. This approach was therefore much more successful than the literature-based selection of reference genes in sunflower [27]. In that report, only one of eight candidates, α-tubulin, displayed comparably low Cq value variability during plant senescence. Furthermore, even applying a combination of all of the tested sunflower genes did not effectively reduce the assay variability in that study (pairwise variation, V = 0.229, above the suggested geNorm threshold). We conclude that the list of candidate reference genes selected in the current study (S1 Table) is a valuable addition to the relatively poor data resources that are available regarding the normalization of senescence-related gene expression experiments in plants.

### Use of reference genes help to reduce the variability across the samples in the ddPCR assays

Similar to qPCR, ddPCR also highly depends on both the sample quality and the accuracy of the reverse transcription step [16]. According to the specification of a popular digital PCR system, QX100 / QX200, the precision of the measurements is +/- 10%. However, to obtain the desired precision, the number of replicates must be sufficiently high. Hindson et al. [15] recently used ddPCR to quantify synthetic oligoribonucleotides that represented 6 mature human miRNAs: miR-16, miR-135b, miR-141, miR-205, miR-210 and miR-375. These authors analyzed the correspondence of the target amount in the samples as calculated from the ddPCR assay to the theoretical amount (250 copies of adequate oligonucleotide per μl PCR). The ddPCR-based measurements corresponded to 114%, 79%, 51%, 49%, 72% and 54% of the theoretically input copies for miR-16, miR-135b, miR-141, miR-205, miR-210 and miR-375, respectively. The ddPCR quantification therefore revealed a two-fold difference between the amount of miR-16 and the amounts of miR-141, miR-205 and miR375, with this effect being due to overall variation of the experiment setup. Such variation likely complicates the biological interpretation of the results. This is an important issue, especially in case of time-course experiments, in which sample-to-sample variation can significantly affect the observed trend of changes in gene expression levels during the studied process.

**Fig 2:**
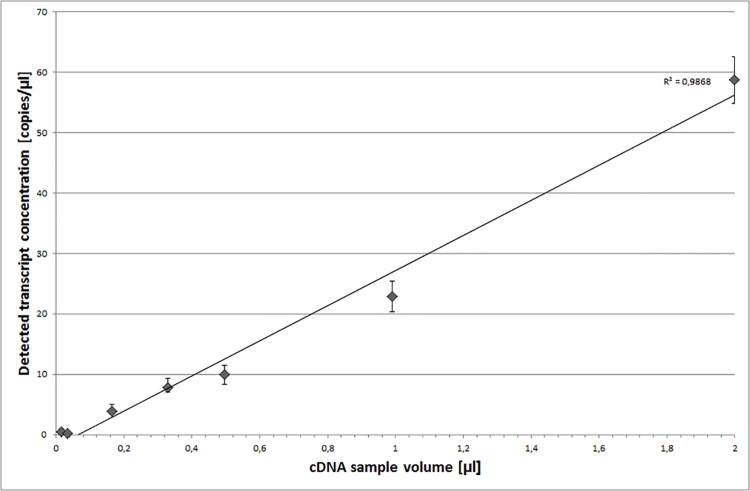
Optimization of the ddPCR sensitivity range and template cDNA input. The presented data are for gene Ref B. The cDNA amount is presented as [μl of RT reaction]. For the assay optimization, the 7-point dilution series (up to 120 x cDNA dilution) was prepared starting from 2 μl cDNA. The error bars indicate the Poisson 95% confidence intervals. The results were similar for all reference genes.

**Fig 3:**
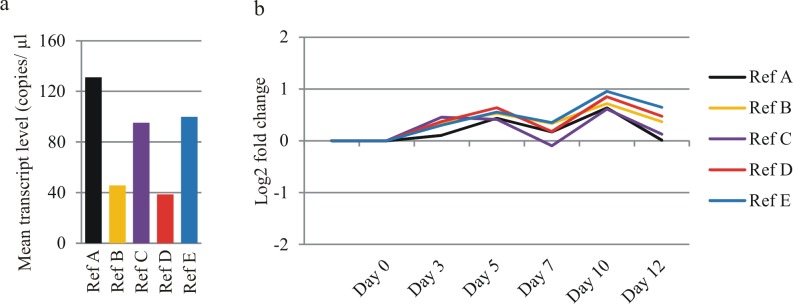
Transcript amount and senescence-associated expression changes of Ref A—Ref E genes in barley leaf. a—Mean transcript amount, averaged from 18 samples (6 time-points × 3 biological replicates); b—Time-course profiles. The data for each time-point were averaged from 3 biological replicates and scaled to Day 0.

**Fig 4:**
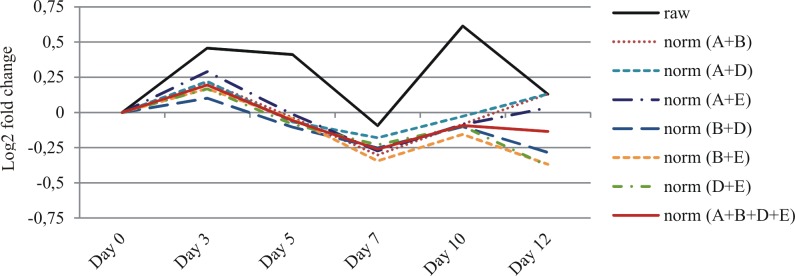
Effect of data normalization on mean Ref C transcript level.

We used ddPCR to quantify the concentration of the Ref A—Ref E transcripts in all of the barley RNA samples. We used the same primer sets as for qPCR because our optimization assays proved that this approach allowed for a clear separation of positive and negative droplet populations (S3 Fig.). Each sample was assayed once, resulting in 3 measurements (corresponding to biological replicates) per time-point. We tested a range of cDNA concentrations and achieved similar sensitivity of our assay as Hindson et al. [15], who reported the detection and quantification of as little as 0.25–2 copies per μl PCR (Fig. 2). We optimized all of the assay conditions to stay well above those limits (S2 Table). We observed that Ref A, Ref C and Ref E were highly expressed, whereas the expression of Ref B and Ref D was 2–3 times lower, on average (Fig. 3A). To evaluate time-course changes of the level of gene expression, series data (from Day 0 to Day 12) were then scaled to Day 0 time point. We observed that the mean concentration of each mRNA was stable throughout senescence, with the maximal change observed at Day 10, which still did not exceed 2-fold (Fig. 3B). The gene expression profiles were very congruent. As they are not functionally related, it is unlikely that this correlation results from the gene co-regulation. We therefore concluded that the main source of variation in our ddPCR assays resulted from intrinsic properties of individual RNA samples, e.g., unequal sample quality or minute changes in template concentration, which may affect the ddPCR assay due to its high sensitivity. This conclusion was clearly supported by data analysis at the level of individual samples (S4 Fig.), suggesting that the reference gene normalization could effectively reduce variation introduced during the experimental steps preceding the ddPCR assays. In agreement with the geNorm-based predictions, any two of the reference genes were sufficient to achieve significant reduction of ddPCR data variation between the biological replicates (Fig. 4, Table 3). Although the coefficients of variation (CV) calculated for the raw data were at least 0.65 in four of six time points, the normalization procedure reduced them below 0.25 (data for the quantification of Ref C gene expression). The normalization was most effective at Day 10 and Day 12. At those time-points, the leaves turn yellow and the cell degradation processes accompanying leaf senescence are in progress, effects that may seriously affect the integrity of RNA isolated from such samples.

**Table 3:** Effect of data normalization on variation of RefC transcript level in ddPCR assays.

ref. gene	none	(A+B)		(A+D)		(A+E)		(B+D)		(B+E)		(D+E)		(A+B+D+E)	
CV	raw	norm	reduce	norm	reduce	norm	reduce	norm	reduce	norm	reduce	norm	reduce	norm	reduce
Day 0	0.73	0.03	**95.2%**	0.09	**87.9%**	0.14	**80.3%**	0.13	**82.6%**	0.16	**78.3%**	0.22	**70.1%**	0.12	**83.2%**
Day 3	0.66	0.17	**74.7%**	0.16	**75.7%**	0.08	**87.5%**	0.21	**68.0%**	0.13	**79.9%**	0.13	**79.8%**	0.15	**77.8%**
Day 5	0.16	0.18	-9.5%	0.04	**75.2%**	0.15	10.0%	0.10	40.3%	0.19	-14.3%	0.08	**51.4%**	0.11	32.0%
Day 7	0.17	0.10	43.1%	0.13	26.4%	0.09	49.7%	0.12	30.7%	0.07	**60.3%**	0.09	46.6%	0.09	45.5%
Day 10	0.65	0.10	**84.6%**	0.08	**88.0%**	0.05	**92.1%**	0.06	**91.1%**	0.05	**92.0%**	0.13	**80.0%**	0.04	**93.7%**
Day 12	0.98	0.23	**76.7%**	0.24	**75.5%**	0.21	**79.0%**	0.11	**89.2%**	0.14	**86.2%**	0.13	**86.8%**	0.05	**95.4%**

Coefficients of variation (CV) were determined by calculating the s.d. from replicates a, b and c (n = 3) and dividing each of these by their respective mean values. Reduction in CV (CV_reduce_) was calculated as the difference between the raw data CV (CV_raw_) and normalized data CV (CV_norm_), divided by raw data CV and expressed as a percentage: CV_reduce_ = (CV_raw_—CV_norm_)/(CV_raw_).

To further evaluate the applicability of reference genes for data normalization, we selected 5 genes that responded similarly to both natural and dark-induced senescence of barley leaf ([26], this work) (S3 Table). Four of these genes were up-regulated: SAG12, encoding a cysteine protease; ICL, encoding isocitrate lyase; AGXT, encoding alanine-glyoxylate aminotransferase; and CS, encoding peroxisomal citrate synthase. The last selected gene, RUBISCO small subunit (RbcS), was strongly down-regulated. Similar expression profiles of selected senescence marker genes were also observed during the natural senescence of Arabidopsis leaves [35], except for ICL, which was very strongly induced in barley but did not show any changes associated with leaf senescing in Arabidopsis.

**Fig 5:**
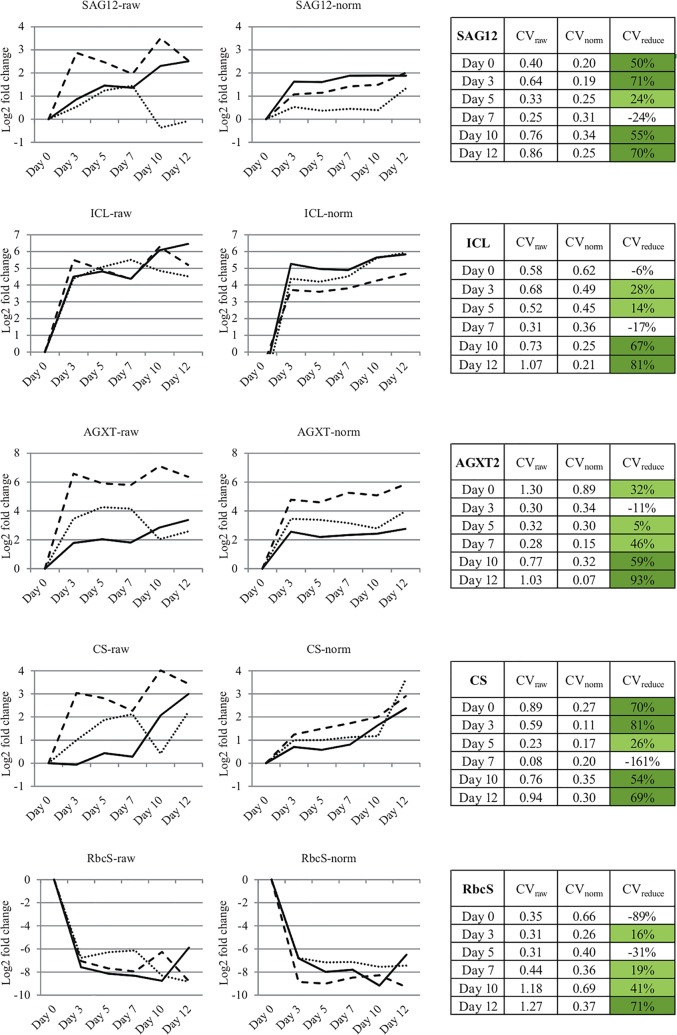
Time-course expression changes of senescence marker genes. The profiles were calculated separately for each biological replicate. The changes are presented in relation to Day 0. The left plots represent raw data, the right plots represent data normalized to geometric mean of Ref A—Ref E genes. The associated tables show the coefficients of variation (CV) for digital PCR assays before (CV_raw_) and after normalization (CV_norm_). The values were determined by calculating the s.d. within each time- point from biological replicates a, b and c (n = 3) and dividing each of these by their respective mean values. The reduction in CV (CV_reduce_) was calculated as the difference between the raw and normalized data CV divided by raw data CV and expressed as a percentage: CV_reduce_ = (CV_raw_—CV_norm_)/(CV_raw_).

We performed ddPCR-based gene expression profiling of the senescence marker genes and analyzed the data by applying normalization factors, calculated as a geometric mean of the Ref A—Ref E transcript levels. After normalization, the variation between biological replicates was substantially reduced. In general, the positive effect of normalization was most apparent for small-fold gene expression changes (as for SAG12 or CS genes). Therefore, the usage of reference genes may contribute to improving the biological interpretation of results in time-course experiments, in which the overall gene behavior must be deduced from the comparative analysis of individual, highly variable measurements (Fig. 5).

## Conclusions

In this study, we generated a list of 181 annotated barley genes with invariant expression during dark-induced leaf senescence. The list is a useful resource for selecting reference genes for senescence-related experiments and supplements the limited available information on this subject in the literature. We note here that the model of dark-induced leaf senescence differs in terms of the expression of some genes that are involved in the natural senescence process [18]. Moreover, the process may be slightly different in distant plant species, e.g., monocots and dicots. The availability of the published microarray gene expression data for natural leaf senescence in barley [26] and Arabidopsis [35] may be helpful in the additional filtering of the candidate genes from our list to meet individual needs.

Our results also highlight the benefits of ddPCR data normalization using well-validated reference genes. Although ddPCR is an absolute quantification method, numerous examples prove that combining it with the use of the reference genes is a valid approach [10–12]. According to our results, the normalization step is highly advisable in cases where the number of repeats is low or the sample quality is expected to be highly variable.

## Supporting Information

S1 Fig Symptoms of dark-induced senescence of barley primary leaf. (PDF)

S2 Fig Expression fluctuations of genes used as stable references in the studies of barley leaf senescence. (PDF)

S3 Fig Separation of positive and negative droplets in ddPCR assays of Ref A—Ref E genes expression. (PDF)

S4 Fig Ref A—Ref E transcript levels in individual samples of barley leaf senescence experiment, measured with ddPCR. (PDF)

S1 Table Genes with stable expression during leaf senescence selected from the barley microarray data (XLSX)

S2 Table Results of ddPCR-based transcript quantification. (DOCX)

S3 Table Microarray data-derived expression changes of well-characterized senescence-associated genes. (DOCX)
