# Supplementary material for: Selection of Reference Genes for qPCR- and ddPCR-Based Analyses of Gene Expression in Senescing Barley Leaves
Source: PLoS One. 2015 Feb 27;10(2):e0118226. doi: 10.1371/journal.pone.0118226 (PMC4344324; doi:10.1371/journal.pone.0118226)
Supplement: S1 Fig — (PDF) [file pone.0118226.s001.pdf]

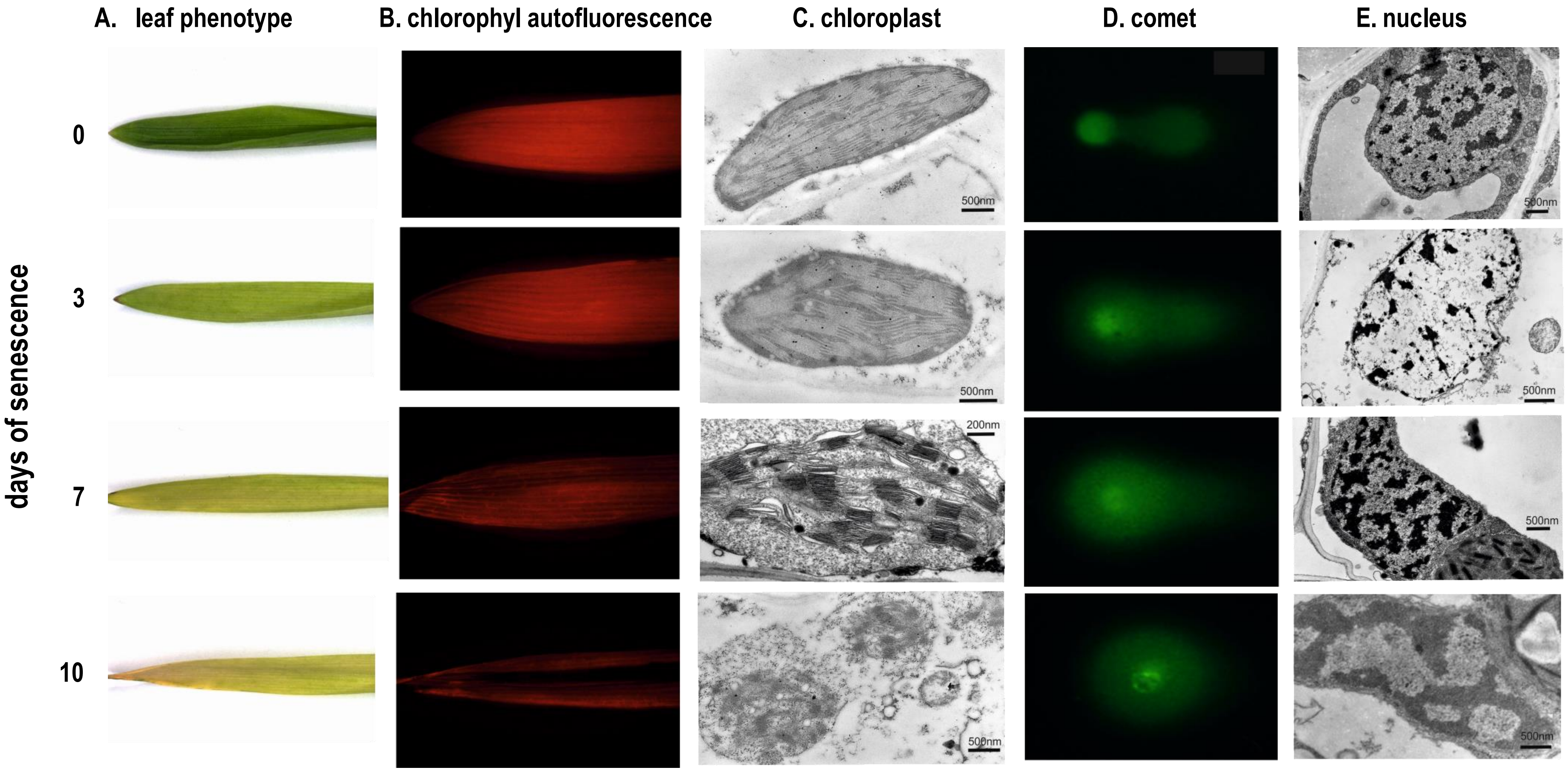

Figure S1. Symptoms of dark-induced senescence of barley primary leaf. Dark-induced senescence is a dynamic process that leads through a series of transformations to the cessation of photosynthesis, the disruption of the organelles and ultimately cell death. **A,** Symptoms of chlorophyll loss in senescing leaves; **B,** Diminishing of the chlorophyll autofluoresence; **C,** decomposition of chloroplast; **D,** internucleosomal fragmentation of chromatin (determined by an alkaline comet assay of the DNA damage); **E** condensation of nuclear DNA and the disruption of nucleus.
