# Supplementary material for: Selection of Reference Genes for qPCR- and ddPCR-Based Analyses of Gene Expression in Senescing Barley Leaves
Source: PLoS One. 2015 Feb 27;10(2):e0118226. doi: 10.1371/journal.pone.0118226 (PMC4344324; doi:10.1371/journal.pone.0118226)
Supplement: S2 Fig — (PDF) [file pone.0118226.s002.pdf]

Expts.	No of treats.	Contig382_s_at	Contig4304_at	Contig2551_s_at	Contig14457_s_at	Contig9163_at	Contig8615_at	Contig8843_at	Experiment Name
BB1	6	5	6	23	9	15	11	13	BB1: Cross-species Detection in Barley1 GeneChip Array
BB2	60	9	4	2	2	4	3	3	BB2: Expression profiling of wild type and mutants of Sultan 5 (Mla12) barley cultivar
BB3	21	9	8	2	3	7	12	7	BB3: Transcription patterns during barley development
BB4	36	4	2	2	1	3	2	2	BB4: Mla-specified Transcriptional Responses in Barley-Powdery Mildew Interactions
BB5	2	1	2	1	0	2	2	0	BB5: A Ca2+/calmodulin-dependent protein kinase required for symbiotic nodule development: Gene identification by transcript-based cloning
BB7	4	7	3	1	2	3	4	3	BB7: mlo5-mediated resistance responses in barley
BB8	11	11	6	10	3	5	10	5	BB8: Microarray of barley genes in wheat-barley chromosome addition lines
BB9	10	13	2	1	1	3	2	2	BB9: Barley cv Morex inoculated with Fusarium graminearum and water as mock control
BB10	48	0	0	0	0	0	0	0	BB10: Transcription profiling of barley plants containing variants of Mla1 and Mla6 powdery mildew resistance genes
BB16	2	5	1	1	1	1	0	1	BB16: Steptoe x Morex seedling leaf comparison
BB20	8	4	2	1	1	3	1	1	BB20: Genotype-dependent gene expression in barley
BB21	37	0	0	0	0	0	0	0	BB21: Genetics of gene expression in barley
BB22	4	9	3	2	1	2	7	1	BB22: Developing seeds of M955 low phytic acid barley 7 days after anthesis
BB28	2	1	1	0	0	0	0	0	BB28: Expression profiling of Morex and rpr1 mutant
BB46	24	13	3	2	3	10	12	4	BB46: Comparison of wild-type and cell death mutant of barley plants containing Mla6 powdery mildew resistance gene
BB47	20	14	6	3	3	5	3	5	BB47: Transcriptome analysis of Bowman vs 4 tillering mutants at 4 developmental stages
BB49	24	5	8	2	5	4	7	5	BB49: barley stem rust interaction
BB50	7	4	3	2	5	6	3	4	BB50: Carbohydrate accumulation in barley leaves leads to senescence and protease gene upregulation
BB52	6	15	2	2	2	2	2	0	BB52: Transcriptome analysis of trichothecene-induced gene expression in barley
BB53	8	6	2	1	4	4	2	5	BB53: Functional Genomic Analysis Of Barley(Hordeum vulgare L.) Grain Protein Accumulation
BB54	5	2	0	2	2	3	2	3	BB54: Necrotic mutants in barley cv. Steptoe
BB55	12	9	3	8	2	3	10	3	BB55: Mapping barley genes to chromosome arms by transcript profiling of wheat-barley ditelosomic chromosome addition lines
BB61	16	2	6	3	5	4	10	4	BB61: Transcriptome analysis of wild barley (H. vulgare ssp spontaneum) after pathogen inoculation
BB62	8	4	1	1	1	3	1	1	BB62: Barley host response to the direct application of the trichothecene mycotoxin deoxynivalenol
BB63	3	1	2	0	1	1	1	0	BB63: Late response to boron toxicity in barley leaves
BB64	154	5	2	1	3	3	3	2	BB64: Genetic regulation of gene expression of barley in response to stem rust (Pgt isolate TTKS)
BB65	10	3	6	4	3	6	5	5	BB65: Transcriptome Analysis of Cold Acclimation in Barley Albina and Xantha Mutants
BB71	8	13	4	2	3	4	3	2	BB71: Microarray analysis of the interaction between Rhopalosiphum padi and partially resistant or susceptible barley lines
BB72	8	4	3	1	2	2	2	2	BB72: Blumeria graminis fsp hordei effects on translation initiation in compatible and incompatible barley.
BB73	4	1	4	0	6	4	2	3	BB73: Comparative transcriptional profiling of organs of the barley spike
BB74	2	0	0	0	0	0	0	1	BB74: Response of barley roots during the host interaction with the plasmodiophorid virus vector Polymyxa graminis
BB75	2	0	1	0	1	0	0	1	BB75: Response of barley roots during the nonhost interaction with the plasmodiophorid virus vector Polymyxa betae
BB76	4	14	1	5	1	5	6	5	BB76: Structural and functional characterization of a winter malting barley
BB77	7	4	1	1	4	2	3	5	BB77: Barley single feature polymorphisms and drought stress gene expression
BB78	8	7	3	1	3	1	7	6	BB78: Array-based genotyping and expression analysis of barley cv. Maythorpe and Golden Promise
BB79	3	11	1	0	0	1	2	2	BB79: P. aeruginosa virulent factor to barley
BB80	3	2	0	1	1	0	1	0	BB80: ABA experiment
BB81	4	1	3	2	2	9	2	2	BB81: Low temperature stress in cv. Dicktoo
BB82	2	15	4	4	5	10	9	5	BB82: Transcriptome analysis of barley anthers: effect of mannitol treatment on microspore embryogenesis
BB83	2	2	2	1	0	1	1	1	BB83: Mercury toxicity in barley roots
BB84	12	4	2	2	4	6	3	2	BB84: Differentially Expressed Genes between Drought-tolerant and Drought-sensitive Barley Genotypes
BB85	8	3	5	1	2	5	6	6	BB85: Expression data from barley maturing grains (Part 1 of 2)
BB86	8	25	5	3	4	4	6	8	BB86: Expression data from barley germinating grains (Part 2 of 2)
BB87	11	13	5	4	3	4	5	8	BB87: Expression data from malting barley seeds
BB88	2	1	1	1	1	0	1	1	BB88: Endophytic colonization of barley (Hordeum vulgare) roots by Pochonia chlamydosporia
BB89	8	2	4	1	5	5	2	5	BB89: Gene expression in the barley spike during drought stress
BB91	30	8	2	5	1	4	6	4	BB91: Transcriptome analysis of a breeding program pedigree
BB92	4	6	2	1	4	1	3	5	BB92: Transcript profiling of local and adjacent leaf responses in barley following inoculation with Pseudomonas syringae
BB93	2	0	0	0	0	1	0	0	BB93: Over-expression of ODDSOC2 in Golden Promise
BB94	4	5	3	1	1	5	4	2	BB94: Short and long term cold responses in a winter barley
BB95	2	3	0	0	1	1	0	0	BB95: Long term cold responses in a winter barley
BB101	12	1	1	3	1	2	2	2	BB101: Transcript profiling of Bln1 silenced plants (BSMV-VIGS) relative to empty vector and buffer treated controls in barley-powdery mildew interactions

Figure S2. Expression fluctuations of genes used as stable references in the studies of barley leaf senescence. The comparison was performed by the PlexDB /GeneOscilloScope tool barley Gene chip data available in that database. Green bars and the values represent CV in % (Coefficients of Variation of the treatment means in the corresponding experiments). Probe sets represent: Contig382_s_at – 18S RNA, Contig4304_at – Splicing factor 2, Contig2551_s_at – Ref A, Contig14457_s_at – Ref B, Contig9163_at – Ref C, Contig8615_at – Ref D, Contig8843_at – Ref E.
