# Supplementary material for: Selection of Reference Genes for qPCR- and ddPCR-Based Analyses of Gene Expression in Senescing Barley Leaves
Source: PLoS One. 2015 Feb 27;10(2):e0118226. doi: 10.1371/journal.pone.0118226 (PMC4344324; doi:10.1371/journal.pone.0118226)
Supplement: S4 Fig — (PDF) [file pone.0118226.s004.pdf]

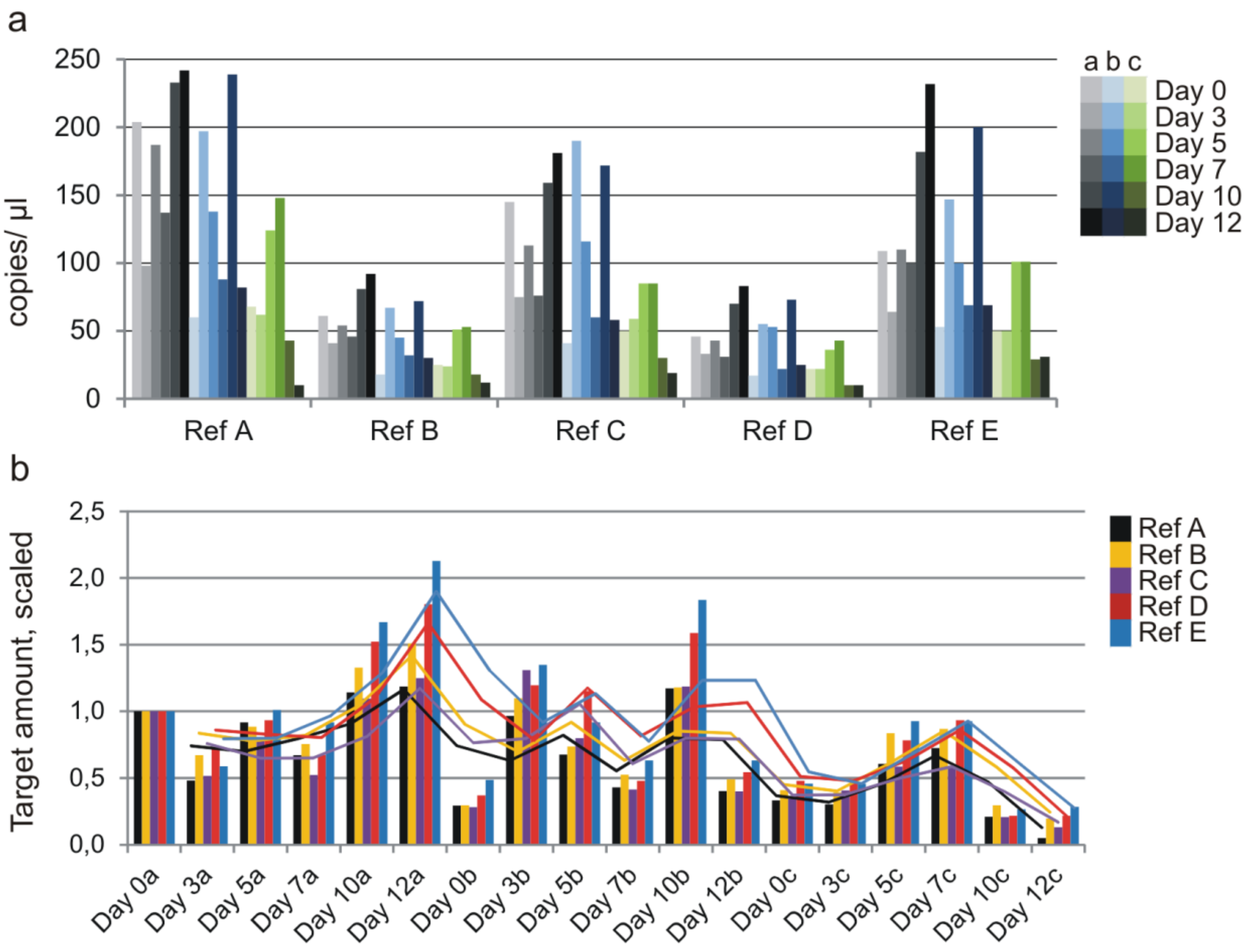

Figure S4. Ref A – Ref E transcript levels in individual samples of barley leaf senescence experiment, measured with ddPCR. a – Calculated number of target copies per µl PCR. Shades of grey, blue and green mark biological replicates a, b and c, respectively. b – The same data scaled to a calibrator sample (Day 0, replicate a). Solid lines reveal similar trends of Ref A – Ref E expression across the samples.
