# Supplementary material for: Selection of Reference Genes for qPCR- and ddPCR-Based Analyses of Gene Expression in Senescing Barley Leaves
Source: PLoS One. 2015 Feb 27;10(2):e0118226. doi: 10.1371/journal.pone.0118226 (PMC4344324; doi:10.1371/journal.pone.0118226)
Supplement: S2 Table — (DOCX) [file pone.0118226.s006.docx]

**Table S2. Results of ddPCR-based transcript quantification.** 1 µg RNA was used as a template in reverse transcription step and 2 µl cDNA was used in each of the following digital PCR assays (20 µl). Absolute transcript amounts in the samples are presented in (copies / µl PCR).

| **Sample** | **Ref A** | **Ref B** | **Ref C** | **Ref D** | **Ref E** | **SAG12** | **ICL** | **AGXT** | **CS** | **RbcS** |
| --- | --- | --- | --- | --- | --- | --- | --- | --- | --- | --- |
| Day 0a | 204 | 61 | 145 | 46 | 109 | 497 | 24 | 226 | 23 | 9600 |
| Day 3a | 98 | 41 | 75 | 33 | 64 | 902 | 541 | 778 | 22 | 50 |
| Day 5a | 187 | 54 | 113 | 43 | 110 | 1362 | 672 | 930 | 31 | 34 |
| Day 7a | 137 | 46 | 76 | 31 | 100 | 1278 | 496 | 792 | 28 | 30 |
| Day 10a | 233 | 81 | 159 | 70 | 182 | 2453 | 1608 | 1620 | 96 | 22 |
| Day 12a | 242 | 92 | 181 | 83 | 232 | 2813 | 2098 | 2335 | 183 | 162 |
| Day 0b | 60 | 18 | 41 | 17 | 53 | 235 | 17 | 8 | 5 | 10700 |
| Day 3b | 197 | 67 | 190 | 55 | 147 | 1718 | 766 | 762 | 41 | 80 |
| Day 5b | 138 | 45 | 116 | 53 | 100 | 1291 | 512 | 480 | 35 | 52 |
| Day 7b | 88 | 32 | 60 | 22 | 69 | 912 | 345 | 442 | 24 | 43 |
| Day 10b | 239 | 72 | 172 | 73 | 200 | 2664 | 1321 | 1093 | 81 | 139 |
| Day 12b | 82 | 30 | 58 | 25 | 69 | 1357 | 622 | 657 | 54 | 24 |
| Day 0c | 68 | 25 | 50 | 22 | 50 | 296 | 6 | 39 | 6 | 5100 |
| Day 3c | 62 | 24 | 59 | 22 | 50 | 429 | 125 | 427 | 12 | 46 |
| Day 5c | 124 | 51 | 85 | 36 | 101 | 700 | 202 | 742 | 22 | 65 |
| Day 7c | 148 | 53 | 85 | 43 | 101 | 801 | 272 | 690 | 26 | 72 |
| Day 10c | 43 | 18 | 30 | 10 | 29 | 229 | 173 | 159 | 8 | 16 |
| Day 12c | 10 | 12 | 19 | 10 | 31 | 280 | 137 | 232 | 28 | 11 |
