# Supplementary material for: Selection of Reference Genes for qPCR- and ddPCR-Based Analyses of Gene Expression in Senescing Barley Leaves
Source: PLoS One. 2015 Feb 27;10(2):e0118226. doi: 10.1371/journal.pone.0118226 (PMC4344324; doi:10.1371/journal.pone.0118226)
Supplement: S3 Table — (DOCX) [file pone.0118226.s007.docx]

**Table S3.** **Microarray data-derived expression changes of well-characterized senescence-associated genes.** Data for natural senescence in Arabidopsis are after [35]; data for natural senescence in barley are after [34]; data for dark-induced senescence in barley come from this work. Gene expression change is presented as log_2_ value.

| Gene | Arabidopsis | | Barley | | | | |
| --- | --- | --- | --- | --- | --- | --- | --- |
|  | Locus | natural senescence | GenBank ID | natural senescence | senescence induced by darkness | | |
|  |  |  |  |  | day 3 | day 7 | day 10 |
| SAG12 | AT5G45890 | up | AK353926 | 1.69* | 1.06* | 1.21* | 1.20* |
| ICL | AT3G21720 | no change | AK252018 | 11.60 | 6.30 | 6.25 | 6.30 |
| AGXT | AT3G08860 | up | AK361069 | 4.50 | 2.89 | 2.80 | 3.03 |
| CS | AT2G42790 | up | AK252959 | 2.82 | 1.26 | 1.30 | 1.61 |
| RbcS | AT4G38970 | down | AK252711 | -2.53 | -7.00 | -6.76 | -7.06 |

* For barley SAG12 gene expression, mean value of 5 perfectly matching probes was calculated
