# Supplementary material for: Selection of Reference Genes for qPCR- and ddPCR-Based Analyses of Gene Expression in Senescing Barley Leaves
Source: PLoS One. 2015 Feb 27;10(2):e0118226. doi: 10.1371/journal.pone.0118226 (PMC4344324; doi:10.1371/journal.pone.0118226)
Supplement: S3 Fig — (PDF) [file pone.0118226.s003.pdf]

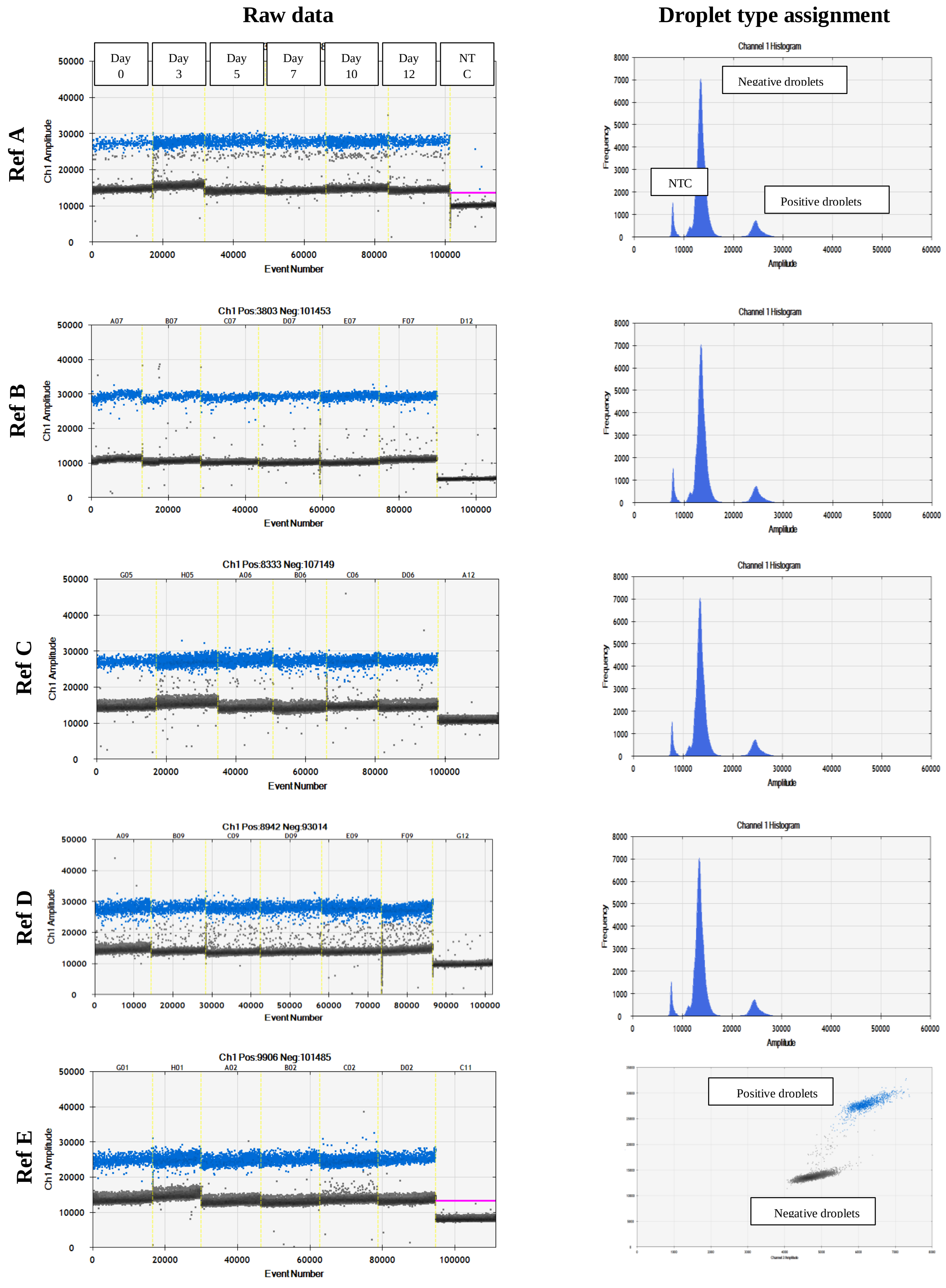

Figure S3. Separation of positive and negative droplets in ddPCR assays of Ref A – Ref E genes expression. Left – droplet plots for biological replicate b (all time points). Right – histogram or two-dimensional plot for one sample (Day X). Droplet populations were detected automatically by the QuantaSoft software (Ref A – Ref D) or set manually from the two-dimensional plot (Ref E). The data is representative for all biological replicates. NTC – no template control
